# Identification of a novel heterozygous *TSC2* splicing variant in a patient with Tuberous sclerosis complex: A case report

**DOI:** 10.1097/MD.0000000000028666

**Published:** 2022-01-21

**Authors:** Linli Liu, Chunshui Yu, Gaowu Yan

**Affiliations:** a Department of Dermatology, Suining Central Hospital, Suining, People's Republic of China; b Department of Radiography, Suining Central Hospital, Suining, People's Republic of China.

**Keywords:** minigene, *TSC2* variant, tuberous sclerosis complex

## Abstract

**Rationale:**

Tuberous sclerosis complex (TSC) is an autosomal dominant genetic disorder characterized by facial angiofibromas, epilepsy, intellectual disability, and the development of hamartomas in several organs, including the heart, kidneys, brain, and lungs. Mutations in either *TSC1* or *TSC2* result in dysregulated mTOR activation, leading to the occurrence of TSC.

**Patient concerns:**

A 44-year-old man was hospitalized for acute lumbago and hematuria.

**Diagnosis:**

The patient presented with facial angiofibromas, epilepsy, fibrous plaques, periungual fibroma, renal angiomyolipomas (AML), pulmonary lymphangioleiomyomatosis (LAM), liver hamartomas, and osteosclerosis. A diagnosis of TSC was made based on clinical manifestations.

**Interventions:**

Next-generation sequencing (NGS) was performed to screen for potential variants, which were verified using Sanger sequencing. The final variant was analyzed using a minigene assay.

**Outcomes:**

A potentially pathogenic novel *TSC2* variant (NM_000548.4, c.336_336 + 15delGGTAAGGCCCAGGGCG) was identified using NGS and confirmed using Sanger sequencing. The in vitro minigene assay showed that the variant c.336_336 + 15delGGTAAGGCCCAGGGCG caused erroneous integration of a 74 bp sequence into intron 4. This novel variant was not found in his unaffected parents or 100 unrelated healthy controls.

**Lessons:**

We identified a novel heterozygous *TSC2* variant, c.336_336 + 15delGGTAAGGCCCAGGGCG, in a patient with classical TSC and demonstrated that this variant leads to aberrant splicing using a minigene assay. Our results extend the understanding of the mutational spectrum of *TSC2*.

## 1 Introduction

Tuberous sclerosis complex (TSC, OMIM #191100 and #613254) is an autosomal dominant genetic disease caused by mutations in *TSC1* or *TSC2*. The morbidity of TSC is nearly 1:10,000 among newborns, and most patients are diagnosed during the first 15 months of life.^[1]^ TSC is characterized by facial angiofibromas, epilepsy, intellectual disability, and development of hamartomas throughout the body, particularly in the brain, skin, heart, and kidneys. Owing to the different locations of lesions, the spectrum of clinical manifestations is very wide among individuals. Some patients are severely affected at an early age and at multiple sites throughout the body. The major causes of mortality in patients with TSC are seizures and renal complications.^[2]^ TSC is believed to have a high spontaneous mutation rate, as suggested by the large number (approximately two-thirds of all cases) of aperiodic cases without a family history.^[3]^*TSC1* and TSC2, which act as tumor growth suppressors and encode the proteins hamartin and tuberin, have been found to be responsible for mTOR overactivation, which may be the underlying mechanism of pathogenesis.^[4]^

The diagnosis of TSC depends mainly on clinical diagnostic criteria.^[5]^ Owing to genetic heterogeneity, the clinical phenotype of the disease presents high variability, thus making the prediction of the phenotype on an individual basis challenging. Even monozygotic twins with the same mutation can exhibit different clinical expressions.^[6]^ The atypical clinical presentation indicates the potential shortcomings of the current diagnostic criteria for TSC.^[7]^ A pathogenic variant of either *TSC1* or *TSC2* detected by genetic analysis represents a separate diagnostic criterion, regardless of clinical findings.^[8]^ With advances in genomic DNA sequencing technology, it is now easy to analyze the entire sequence of TSC genes. However, molecular diagnosis of TSC remains challenging because of the difficulty in correctly assessing gene variants of unknown significance. A functional analysis is required to determine the pathogenicity of these variants.

Here, we present the clinical manifestations of a patient with TSC and detected a novel *TSC2* gene variant, c.336_336 + 15del16, by Next Generation sequencing (NGS) and Sanger sequencing. An in vitro minigene assay was performed to investigate the effect of this variant on the splicing process.

## 2 Ethics and methods

Written informed consent was obtained from the individual and approval of the Institutional Medical Ethics Committee of Suining Central Hospital. Genomic DNA was extracted from the peripheral blood samples of the patient. After quality control of the extracted DNA samples, a targeted NGS panel (Illumina HiSeq2000 platform, Inc., San Diego, CA, USA) was used to analyze the exons and flanking intronic regions of the *TSC1* and *TSC2* genes associated with TSC. The sequencing data were aligned to the human reference genome hg19 (University of California Santa Cruz (UCSC, http://genome.ucsc.edu). Sanger sequencing was performed to verify the NGS results. Primers were designed using PRIMER 5 software for PCR amplification, according to the potential pathogenic variants obtained by NGS. After purification, the PCR products were sequenced using an ABI PRISM 3730 Genetic Analyzer. Finally, a minigene assay was performed to study the effects of the variants at the transcript level. Mutant and wild-type pMINI-*TSC2* vectors were transfected into 293T cells. After transfection for 48 hours, the total RNA was extracted and reverse-transcribed into cDNA. Following, the cDNA was amplified by PCR, analyzed by agarose gel electrophoresis, and sequenced.

## 3 Case presentation

**Figure 1:**
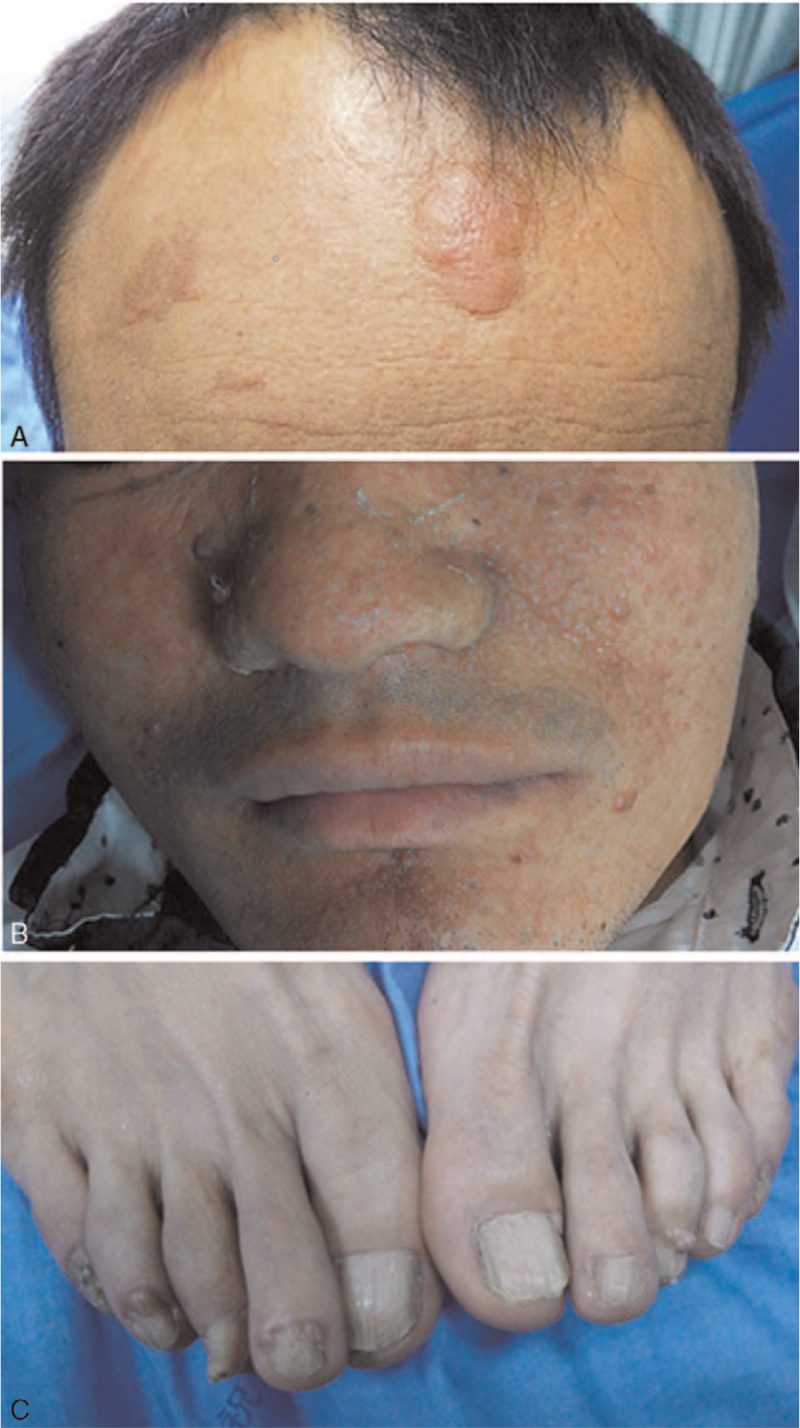
Clinical photographs of the patient with TSC. (A) Fibrous plaques, (B) facial angiofibromas, and (C) periungual fibromas (TSC = tuberous sclerosis complex).

**Figure 2:**
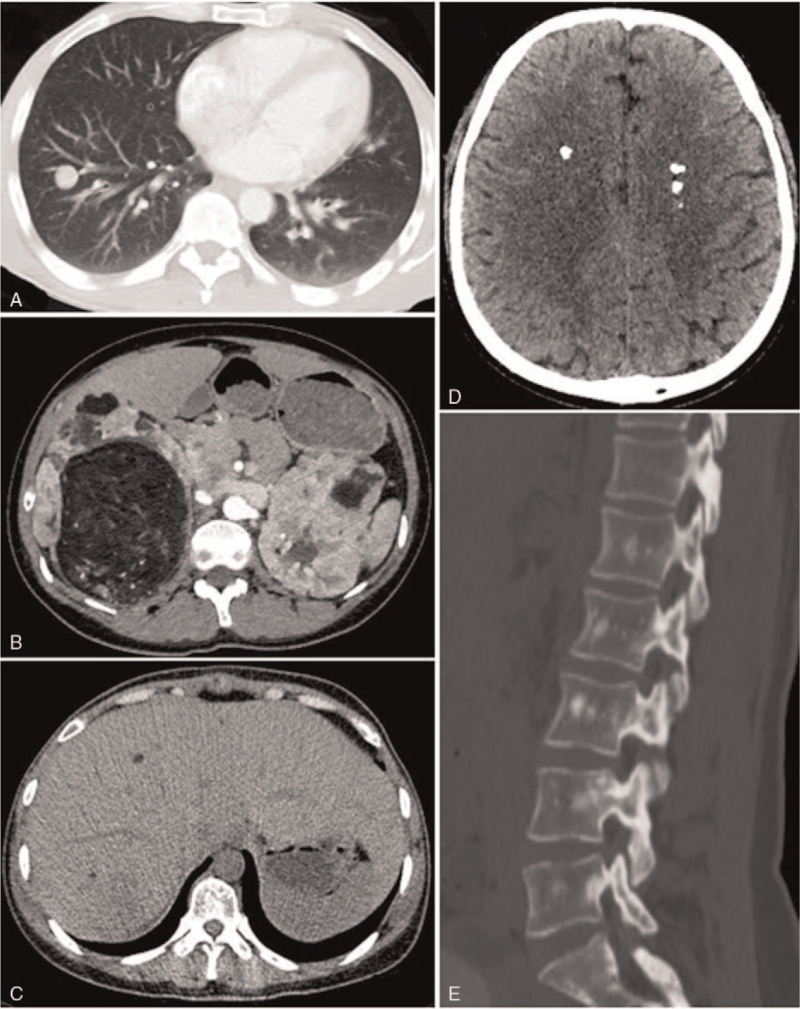
CT Imaging of the patient with TSC. (A) Lung CT scans showing presence of lymphangiomyomatosis, (B) abdomen CT scans showing angiomyolipomas, (C) liver CT showing small hamartomas, (D) brain CT showing multiple calcified subependymal nodules, and (E) spine CT showing multiple patchy sclerotic lesions (TSC = tuberous sclerosis complex and CT = computed tomography).

A 44-year-old man from the Han dynasty presented with a chief complaint of “acute lumbago and hematuresis” for three days and was admitted to the urology department of our hospital. He had a long history of seizures and was being treated with antiepileptic medication. Facial angiofibromas, fibrous plaques, and multiple periungual fibromas were discovered on physical examination (Fig. 1). Biochemical laboratory tests revealed renal insufficiency and moderate anemia. Non-contrast-enhanced computed tomography (CT) of the abdomen revealed large renal AMLs with prominent fatty components and prominent internal vessels bilaterally. Lung CT and consistent lymphangioleiomyomatosis (LAM) revealed multiple air-filled cysts of variable sizes bilaterally. Small hamartomas were noted on liver CT. Multiple calcified subependymal nodules were observed on the brain CT. Spinal CT revealed multiple patchy sclerotic lesions (Fig. 2). No treatment had been administered in the past. There was no family history of similar diseases. Ultimately, we concluded that the patient fulfilled the diagnostic criteria for TSC.^[5]^ Owing to the ruptured hemorrhage of his renal AMLs, the patient eventually underwent surgery. After year of follow-up, the patient remained stable.

**Figure 3:**
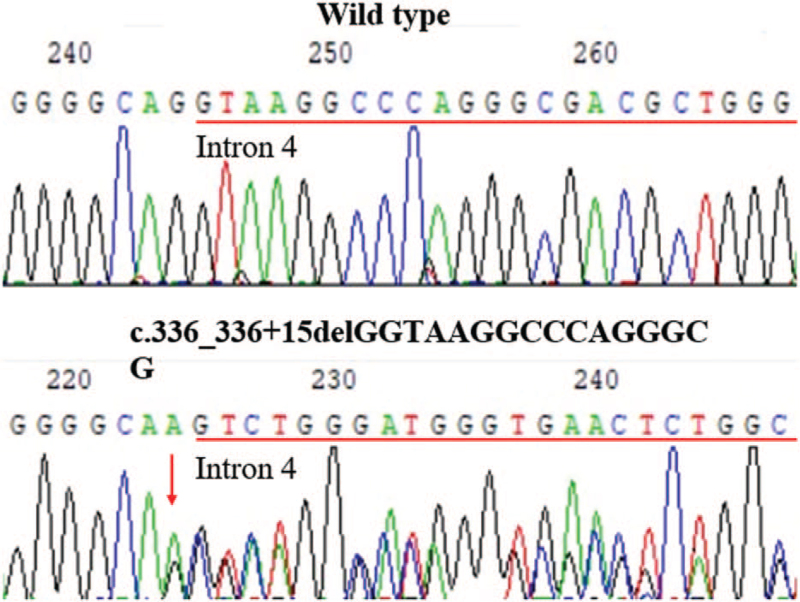
*TSC2* variants identified in the patient with TSC (TSC = tuberous sclerosis complex).

**Figure 4:**
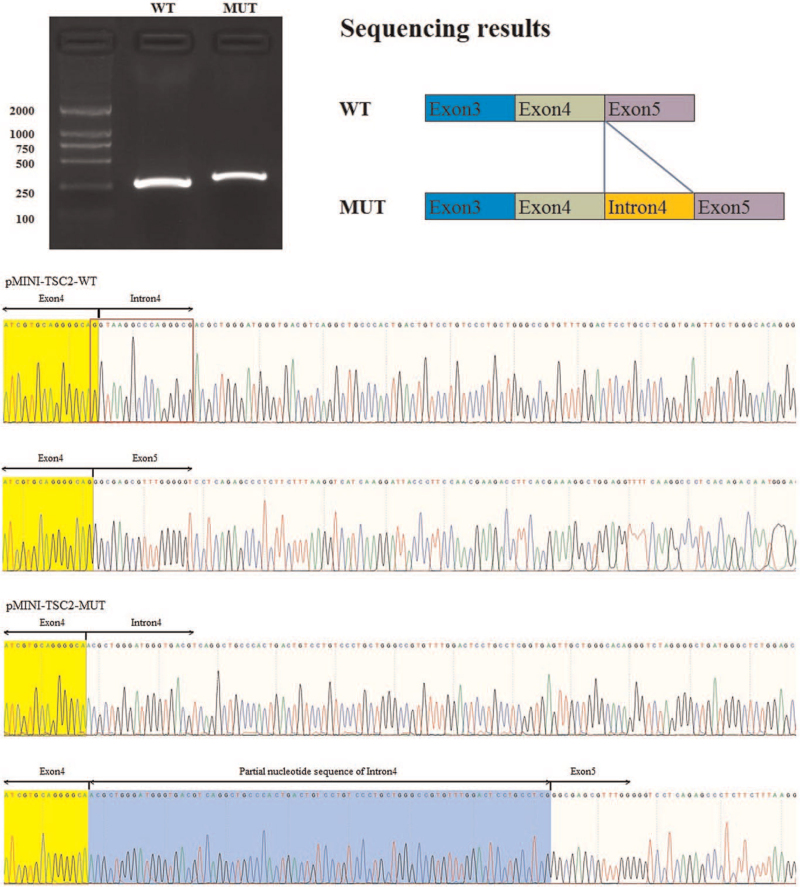
Transcript analyses of pMINI-*TSC2.* Electrophoresis showed that the variant c.336_336 + 15del16 led to a subtly larger *TSC2* mRNA transcript than the wild-type clone. Sequencing showed that the pMINI-*TCS2*-WT was considered as expected normal splicing, while the mRNA sequence of pMINI-*TCS2*-MUT was changed, the original splicing site was lost and erroneous insert of a 74 bp sequence into intron 4, making the splicing position moved back 90 bp (r.336delins336 + 16_336 + 90) (the diagrams above and below represent the plasmid sequencing and mRNA sequencing, respectively).

Next-generation and Sanger sequencing revealed a novel heterozygous TSC2 splice variant (NM_000548.4, c.336_336 + 15del16). This variant was not found in his family members or in the 100 unrelated controls (Fig. 3). This was considered novel because it was not present in the ExAC, 1000G, or HGMD databases. It is noteworthy that the *TSC2* variant c.336_336 + 15del16, resulting in the deletion of the last nucleotide G in exon 4 and the subsequent 15 bases in the intron region, is more likely to cause a complete loss of the splice donor site of intron 4. This variant was more likely to cause splice defects. Minigene assay electrophoresis showed that the c.336_336 + 15del16 variant led to a slightly larger *TSC2* mRNA transcript than that in the wild-type clone. Sequencing showed that pMINI-*TCS2*-WT was considered to have normal splicing, whereas the mRNA sequence of pMINI-*TCS2*-MUT was changed, the original splicing site was lost, and erroneous insertion of a 74 bp sequence into intron 4 caused the splicing position moved back 90 bp (r.336delins336 + 16_336 + 90) (Fig. 4). This variant led to aberrant splicing, and this change was predicted to be p.Gly113Argfs∗5. Based on classification standards and guidelines for ACMG genetic variation, the c.336_336 + 15del16 variant was classified as pathogenic (PVS1 + PS2 + PM2).

## 4 Discussion

TSC is an autosomal dominant neurocutaneous disorder affecting multiple organs. Two genes involved in TSC have been identified; the *TSC1* gene coding for hamartin is located on chromosome 9q34, whereas the *TSC2* gene coding for tuberin is located on chromosome 16p13.3.^[1]^ Mutations in either *TSC1* or *TSC2* result in dysregulated mTOR activation, leading to the occurrence of TSC. In general, *TSC2* variants cause more severe phenotypes than *TSC1* variants.^[9]^ TSC manifests in many organ systems including the brain, skin, heart, kidneys, and lungs. Approximately 80% of TSC cases are associated with renal involvement, such as angiomyolipomas (AML) and polycystic kidney disease (PKD). In such patients, PKD is frequently associated with *TSC2/PKD1* contiguous gene syndrome.^[10]^ The central nervous system is almost invariably involved, with up to 85% of patients presenting with epilepsy, and at least half of the patients have intellectual disabilities or other neuropsychiatric disorders, including autism spectrum disorder.^[11]^ In this study, the patient was characterized by classical multisystematic symptoms, including severe renal AMLs, pulmonary LAM, multiple calcified subependymal nodules, liver hamartomas, skin impairment, and osteosclerosis, indicating a more severe grade of illness. The patient eventually underwent surgery because of rupture hemorrhage of the renal AMLs.

In this study, using NGS and Sanger sequencing of TSC genes, a novel heterozygous *TSC2* variant c.336_336 + 15del16 of exon 4 was identified. This variant leads to deletion of the last nucleotide G in exon 4 and the subsequent 15 bases in the intron region. Notably, this position may play an important role as a splicing modulator that may alter the splicing of the *TSC2* transcript. The minigene splicing assay confirmed that the variant c.336_336 + 15del16 led to the loss of the original splicing site and erroneous insertion of a 74 bp sequence into intron 4, causing the splicing position moved back 90 bp (r.336delins336 + 16_336 + 90). This variant may disturb normal splicing alterations during splicing. Therefore, this variant might lead to a defective tuberin and severe impairment of the normal hamartin–tuberin interaction, thus resulting in upregulation of the mTOR pathway, which in turn might cause the disease. Notably, splice mutations may play a more important role in human hereditary diseases.^[12]^ Classical splicing variants that affect nucleotides at the splice acceptor and donor sites lead to aberrant RNA splicing. However, all types of variants (missense, nonsense, and small insertions or deletions) can lead to splicing defects either by disrupting or creating signals. Zhang et al^[13]^ reported a missense mutation, c.3610G > A, in which the last nucleotide of exon 29 in *TSC2* was replaced. This leads to the substitution of a single amino acid from glycine to arginine at amino acid position 1204 (p. Gly1204Arg), affecting normal splicing. Qiu^[14]^ reported a novel *TSC1* frameshift mutation (*TSC1* c.1550_1551del) that triggered aberrant splicing simultaneously, leading to TSC formation. Additionally, the in vitro minigene assay is an attractive alternative for assessing the impact of intronic variants on splicing.

The c.336_336 + 15del16 variant was considered a novel variant because it was not found in the ExAC, ESP, 1000G, and HGMD databases and was absent in his parents and 100 healthy controls. According to the classification standards and guidelines of ACMG genetic variation,^[15]^ the c.336_336 + 15del16 variant was considered as the LOF variant (loss of function), which conforms to very strong disease-causing evidence (non-functional variant, splice site variants), strong pathogenicity evidence (the patient had a de novo mutation and no family history), and moderate pathogenicity evidence (the mutation was not found in ExAC, ESP, 1000G, HGMD database), namely PVS1 + PS2 + PM2, which was considered pathogenic.

In summary, we highlighted the clinical features of patients with classical TSC. DNA sequencing and minigene splicing assays showed that the novel splicing variant c.336_336 + 15del16 within exon 4 and intron 4 of the *TSC2* gene is a pathogenic variant. This variant affects mRNA splicing, leading to loss of tuberin function, which may be the underlying cause of TSC. The identified variant extended the *TSC2* mutational spectrum and, to some extent, increased our understanding of the molecular mechanism of TSC pathogenesis.

## Acknowledgments

We thank the patient who agreed to use the images and clinical data.

## Author contributions

**Data curation:** Linli Liu, Gaowu Yan.

**Funding acquisition:** Chunshui Yu.

**Writing – original draft:** Linli Liu.
